# Efficacy of Phytopharmaceuticals From the Amazonian Plant *Libidibia ferrea* for Wound Healing in Dogs

**DOI:** 10.3389/fvets.2020.00244

**Published:** 2020-06-12

**Authors:** Ádria Vanessa Linhares dos Santos Américo, Kariane Mendes Nunes, Francisco Flávio Vieira de Assis, Salatiel Ribeiro Dias, Carla Tatiane Seixas Passos, Adriana Caroprezo Morini, Junior Avelino de Araújo, Kelly Christina Ferreira Castro, Silvia Katrine Rabelo da Silva, Lauro Euclides Soares Barata, Antonio Humberto Hamad Minervino

**Affiliations:** ^1^Laboratory of Animal Health (LARSANA), Federal University of Western Pará, Santarém, Brazil; ^2^Laboratory of R&D on Pharmaceutical and Cosmetic, Federal University of Western Pará, Santarém, Brazil; ^3^Bioactive Research and Development Laboratory, Federal University of Western Pará, Santarém, Brazil; ^4^Microbiology Laboratory, Federal University of Western Pará, Santarém, Brazil

**Keywords:** open wound, Carbopol gel, murumuru fat, Jucá, antimicrobial activity

## Abstract

We comparatively evaluate two distinct formulations containing 5% of Jucá (*Libidibia ferrea*) for wound healing in dogs. An excision model study was performed in 11 dogs with three dermal wounds in each animal, which were treated with: (1) topical phytopharmaceutical based on Carbopol (PyC) containing 5% Jucá ethanolic extract; (2) topical phytopharmaceutical based on *Astrocaryum murumuru* butter (PyM) containing 5% Jucá ethanolic extract; and (3) commercial ointment (control). Wound treatment was carried out on alternated days starting at day (D) one until D21. Macroscopic (all time-points) and histological (D0 and D21) analyses were performed. The antimicrobial activity of Jucá was evaluated through Minimal Inhibitory Concentration (MIC). Phytochemical analysis of Jucá revealed 3.1% phenolic compound content expressed in rutin and the presence of hydrolyzable tannins and flavonoids. The mean wound retraction was 33.7 ± 5.5, 34.0 ± 4.7, and 28.4 ± 4.9 % for PyC, PyM, and control groups, respectively, with higher wound retraction for both herbal-treated groups compared to the control (*P* < 0.05). Alcoholic extract of Jucá had antimicrobial activity against the microorganisms *Staphylococcus aureus, Escherichia coli, Pseudomonas aeruginosa*, and *Candida krusei* at different degrees, with MIC ranging from 250 to 16.625 μg/ml. Microscopic evaluation showed that the phytotherapic formulations contributed to better dermal wound healing through wound fibroplasia. The alcoholic extract of Jucá pods has great potential for wound healing in dogs and can be used in the development of commercially viable phytotherapic formulations.

## Introduction

The use of plant products for wound healing has been extensively studied (1, 2) including in veterinary medicine (3, 4) but there are limited reports dealing with native plants from the Amazon. *Libidibia ferrea (*Mart. ex Tul.) var. ferrea, is a Brazilian tree found mainly in the north and northeast regions, and is commonly known as *Jucá* or *pau-ferro* (5, 6). In the Amazon, Jucá is extensively used in popular medicine to treat various health conditions, including bronchopulmonary diseases, diabetes, rheumatism, cancer, gastrointestinal disorders, and diarrhea, in the form of tea and infusions. It is also used for the topical treatment of wounds and contusions (7, 8). In folk medicine of the Lower Amazon, whole pods of the Jucá are immersed with alcohol and used for healing a variety of dermal wounds.

The diverse biological properties of Jucá have been extensively investigated, including its anti-inflammatory, analgesic, anticancer, antioxidant, and antimicrobial effects (9–12). There are also studies on the phytochemical composition of Jucá showing the presence of phenolic compounds, flavonoids, and tannins (5, 13).

Despite the fact that Jucá pods (fruits) are widely used for wound healing in the Amazon region, limited scientific literature is available. There are reports of wound healing properties of different parts of the plant (such as seeds and bark), in goats, (7) donkeys (14), and Wistar rats (15). Kobayashi et al (16), using two ethanolic extracts (12.5 and 50%) of Jucá pods for wound healing in rats, surprisingly found that the negative control was more efficient for wound retraction.

Studies with extracts obtained from the ethanolic extract from Jucá pods for the treatment of wounds, similar to those carried out empirically in the Amazon, are scarce, and no study has been performed on dogs, a species that has a high demand for products with dermal wound healing properties Skin lesions in dogs are frequent and have many causes. There is no herbal formulation on the market in Brazil for dermal wounds. Thus, we aimed to evaluate the wound healing properties of two different semi-solid formulations containing a lyophilized ethanolic extract of Jucá (*L. ferrea*) in dogs.

## Materials and Methods

### Plant Collection and Identification

Leaves, fruits, and flowers of an adult Jucá plant from urban area of Santarém - PA, (2°25'20 “S, 54°42'14” W) were collected and deposited in the HSTM Herbarium at the Federal University of the West of Pará (UFOPA), where it was cataloged with exsiccata number HSTM010436, and identified as FABACEAE, *Libidibia ferrea (*Martius Ex Tulasne) L. P. Queiroz var. ferrea. The fruits used to produce the crude extract were collected in June and July of 2016.

### Extraction and Chemical Fingerprinting

The ethanolic extract of Jucá was obtained according to the methods described elsewhere (16). Briefly, 1.5 kg of the fruit was cleaned with alcohol 70% GL, dried at room temperature for 48 h, and dried in a forced circulation air oven at 55°C for 72 h. The pods were ground in a knife mill (<1 mm) and macerated with 96% GL ethanol, at the rate of 5 L of solvent to 1 kg of the pod. The ethanolic solution was kept at room temperature for 7 days and was then filtered through Whatman's No. 1 filter paper and subsequently concentrated using a rotary evaporator. Finally, the extract was lyophilized at 55°C and stored at −20°C until being used for chemical and biological analyses and in the preparation of the herbal formulations.

The chromatographic profile of the phenolic compounds found in the dried extract of Jucá was obtained by thin-layer chromatography (TLC). A 30 mg/mL ethanolic extract sample was used as the stationary phase and the elution system was composed of ethyl acetate:methanol (9:1) and dichloromethane (*v/v*). Tannins and flavonoids were detected with solutions of 5% ferric chloride and 5% aluminum chloride. The reference standards used for ultraviolet (UV) detection were green tea for the tannin class and rutin for the flavonoid class (17). For chromate plate readings, a darkroom with translucent UV light (254 nm) was used.

The total phenolic compound content was determined as described elsewhere (18) using a flavonoid as a routine standard. The results were obtained by generating a calibration curve (350 nm) of the standard solution at concentrations of 2, 4, 8, 10, 20, 40, and 60 μg/mL, all in triplicate. For the preparation of the standard solutions and the Jucá extract, 100% methanol was used as the solvent. An aliquot of the lyophilized Jucá extract was dissolved in methanol to obtain a concentration of 50 μg/mL.

### Animal Husbandry

Eleven mongrel dogs were used, weighing between 5 and 25 kg. Health status was confirmed by physical examination, blood count, and a negative Leishmaniasis test, since this disease is endemic in the region (19). The animals were from the Center for Zoonoses Control (CZC), located in the municipality of Santarém, and were kept in individual cages (1 × 2 m) at the CZC with water and feed *ad libitum* throughout the study. The experiment followed the regulations for the use of animals in scientific experimentation and was approved by the Committee on Ethics in the Use of Animals (CEUA) under Protocol No. 0120180006. The chosen number of dogs (11) was defined considering (I) the minimal number for an appropriate statistical analysis, (II) adequate animal heterogenicity (small, medium and large dogs), and (III) a limited number of dogs were used due to animal welfare concerns.

### Wound Healing

#### Ointments and Animal Groups

In the experimental design, two semisolid topical formulations containing Jucá ethanolic extract were selected: topical phytopharmaceutical based on Carbopol® (PyC) and topical phytopharmaceutical based on *Astrocaryum murumuru* butter (PyM). In the preparation of both formulations, the constituents were heated to 45°C under constant manual stirring, and the lyophilized Jucá extract (5%) was incorporated using propylene glycol as a leavening agent. As the formulation base, we used Carbopol® 940 gel (PyC), and a crystalline liquid system, obtained as described in the patent application BR 1020150308884, with appropriate amounts of *Astrocaryum murumuru* butter (PyM). Formulation compositions are presented in Table 1. The inclusion of an additional group (negative control), treated only with the ointment base, was not possible in this study due to animal welfare and legal issues. We decided to use a 5% concentration due to prior results obtained in cattle from our research group that established 5% as the best concentration for dermal wound healing (20).

**Table 1:** Composition of the semi-solid phytotherapic formulation containing lyophilized Jucá extract (*Libidibia ferrea*).

**Jucá formulations**	**Murumuru butter (%)**	**Carbopol^®^ (%)**	**Surfactant (Procetyl^®^) (%)**	**Lyophilized extract of Jucá (%)**	**Propylene glycol (%)**	**Water (%)**
PyM	40	0	40	5	5	10
PyC	0	1	–	5	5	100 q.s.p

*PyM, Phytopharmaceutical based on murumuru butter; PyC, Phytopharmaceutical based on Carbopol*.

After 24 h of preparation, the phytopharmaceutical based on murumuru butter was evaluated by polarized light microscopy (PLM) in order to verify the formation of the crystalline liquid phase. The phytopharmaceutical based on Carbopol® was subjected to the centrifugation test.

In each of the 11 animals, three dermal wounds were surgically made, and each wound was treated with a different phytopharmaceutical (murumuru butter or Carbopol gel) or with a commercial veterinary ointment (control), with allantoin (3.0 g) and zinc oxide (3.0 g) (Alantol, Vetnil, São Paulo, Brazil).

The animals were followed for 21 days with assessments at the beginning of the study, immediately after making the wounds (D0), and after 1 (D1), 4 (D4), 7 (D7), 14 (D14) and 21 (D21) days. Each wound was assessed individually with photographic recordings and macroscopic evaluations at all pre-established time-points. Hematological evaluations were performed at all time-points and histological analyses were performed on D0 and D21.

### Wound Incision and Ointment Application

**Figure 1:**
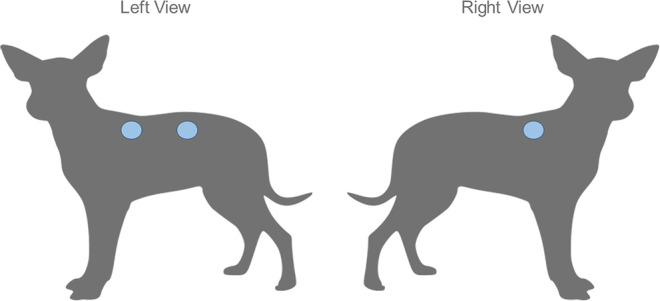
Anatomical disposition of the standardized dermal wounds in dogs. Three standardized dermal wounds induced in the dorsal region of each animal between the first thoracic vertebra and the fifth lumbar vertebra, 5 cm from the cervical spine, and 10 cm apart, two in the left side and one in the right side.

The excision wound model was used. Three standardized dermal wounds were surgically made in the dorsal region of each animal between the first thoracic vertebra and the fifth lumbar vertebra, 5 cm from the cervical spine, and 10 cm apart (Figure 1). Standardized dermal wounds were performed after trichotomy under sterile conditions. Local anesthesia with 2% lidocaine was used. Three fragments of cutaneous tissue were removed from each dog using a 10 mm diameter biopsy punch with metal delimiter to standardize the depth to 5 mm.

Each wound was treated (starting at D1) exclusively with a single ointment every 36 h in the first 7 days and every 48 h from day 8 to 21, totaling 12 topic treatments. For ointment application the site was initially cleaned with a 0.9% NaCl solution followed by the manual application of ~0.1 mL of each ointment (PyM, PyC and control) directly on the respective lesions. Treatment was distributed using a caster system to avoid possible interference between the lesion site and the healing process (i.e.,: the wound location chosen for each treatment was sequentially alternated between the experimental animals).

### Wound Evaluation

Macroscopic evaluation of each wound was done by checking for the presence or absence of exudate, crusts, epithelization, flies, and fly larvae around or inside the wound. The wound healing was observed after 21 days of treatment and classified as normotrophic (texture and consistency similar to baseline) or hypotrophic/hypertrophic (tissue with inadequate texture with non-harmonic aspect).

The lesions were photographed at all time-points and evaluated using a digital camera affixed to a stand, in order to maintain a standardized distance. The images of the wounds were analyzed using the digital morphometry software (ImageJ®) to obtain the diameter of each wound at each time-point. The wound contraction was calculated according to the formula described by Oliveira et al. (7): CI = 100 × (W0–WI)/W0, where: CI: contraction index (%), W0: initial wound area (D0), WI: final area (D14 or D21).

For microscopic evaluation skin biopsies, samples were obtained at D0 and D21. Skin samples were fixed in a 10% formalin solution and stained with hematoxylin and eosin, and visualized under an optical microscope (200×, 100×, and 50×). Histochemical analysis was performed on tissue cross-sections using the Gomori Trichrome staining to identify the cicatrization phase: inflammatory, proliferative, or maturation.

Microscopical evaluation considered the collagen intensity, evaluated by the amount of collagen fibers; intensity of the inflammatory response, evaluated by the amount of macrophages and neutrophils at the site of injury; vasodilation, evaluated by the amount of fibroblasts present per field. Microscopical analysis allowed us to qualitatively evaluate the histomorphological differences between the wounds of the same animal and to compare the healed tissue (at D21) with the heathy tissue (D0). All analyses were made using an optical microscope by the same pathologist.

### Antimicrobial Activity

*In vitro* antimicrobial activity was determined by the microdilution method in a 96-well plate, using the M27-A2 protocol of the Clinical and Laboratory Standard Institute (21) to determine the minimum inhibitory concentration (MIC), expressed in micrograms per milliliters (μg/mL). Gram-positive bacteria *Staphylococcus aureus* (ATCC14458), and Gram-negative bacteria, *Escherichia coli* (ATCC 25922), *Pseudomonas aeruginosa* (ATCC 19429) were cultured in Muller Hinton Agar (Sigma-Aldrich), and the fungus *Candida krusei* (ATCC 6258) in Sabouraud Dextrose Agar (Neogen medium). Microbial suspensions were adjusted to the 0.5 MacFarland scale with 0.9% saline solution equivalent to 108 colony-forming units (CFU) per mL for bacteria and 104/mL spores for fungi.

The antimicrobial activity of the lyophilized extract of the jucá (*Libidibia ferrea*) was evaluated at concentrations ranging between 1,000 and 15.625 μg/mL. The antibiotic ciprofloxacin (5 μg/mL) was used as a positive control for the antibacterial test and nystatin (64 μg/mL) for the antifungal test. The test was carried out for 24–48 h at 37°C and, subsequently, they were revealed in a solution of 10 μL of 0.01% resazurin as a colorimetric indicator to characterize cell viability in each well of the test plate.

In order to elucidate the antibacterial properties (bactericidal or bacteriostatic) of jucá, a loopful of aliquots from the MIC wells was transferred onto the respective culture medium (for fungus or bacteria) and incubated for 24–48 h. If bacteria/fungus failed to resume growth during incubation, the jucá concentration was considered to be bactericidal/fungicidal, otherwise, it was bacteriostatic/fungistatic.

### Statistical Analysis

The data from wound diameter and wound retraction was submitted for analysis of variance using the Generalized Linear Model (GLM) considering the fixed factors day, treatment (PyM, PyC and control) and animal. Means were compared through Fisher Least Significative Difference (LSD) method. The hematological variables were analyzed by paired ANOVA to evaluate the effect of time. A chi-square test was used to evaluate the outcomes of macroscopic evaluation considering the presence or absence of clinical alterations (hyperemia, exudate, edema, crusting, and wound healing). The analyses were performed using Minitab 17 (Minitab Inc., State College, USA) statistical software. Statistical significance is set at *p* ≤ 0.05.

## Results and Discussion

### Chemical Analysis

**Figure 2:**
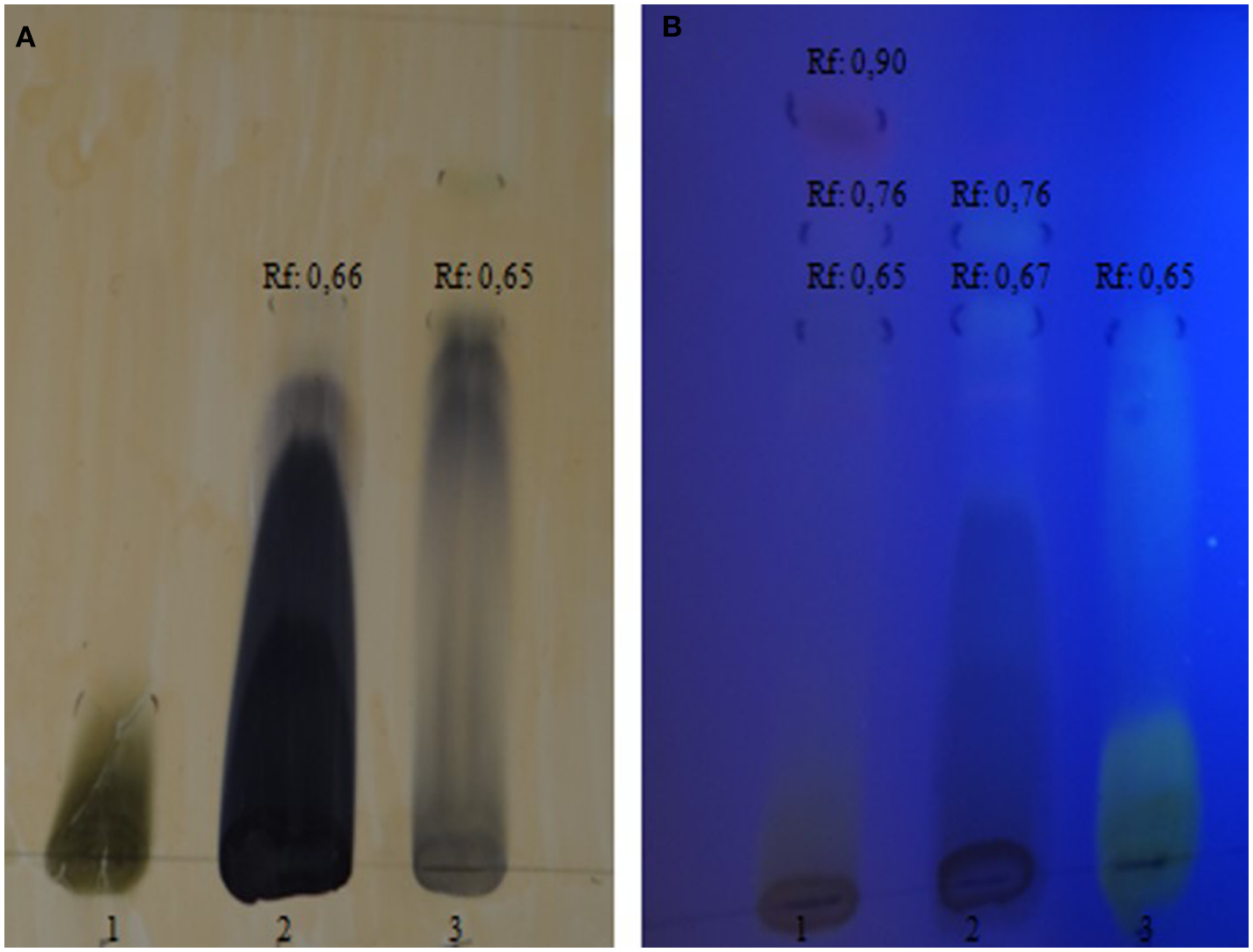
Chromatographic profile of the ethanolic extract from *Libidibia ferrea* obtained by thin layer chromatography. **(A)** hydrolysable tannins in visible light; 1: rutin standard; 2: tested extract; 3: green tea standard; **(B)** flavonoids in ultraviolet light; 1: green tea standard; 2: tested extract; 3: rutin standard.

The preliminary chemical characterization of the phenolic compounds identified in the ethanolic extract of Jucá by TLC showed a significant amount of hydrolysable tannins and flavonoids. The extract showed a dark blue spot with a retention factor (Rf) of 0.66, characteristic of hydrolysable tannins, and a light-yellow spot (Rf = 0.67), characteristic of flavonoids (Figure 2). The reference standards for tannins and flavonoids had a Rf equal to 0.65. Similar results were also found in previous analyses of ethanolic and methanolic extracts of *L. ferrea* (13, 16).

The total concentration of phenolic compounds was determined using a linear equation based on the calibration curve Abs (nm) = 0.0265C (μg / mL) + 0.1306, R^2^ = 0.993, which exhibited a 3.1 % content of rutin phenolic compound in 50 mg of the extract. The phenolic compound of Jucá varies both in the analytical method used and the part of the plant studied (5). The most commonly used method is the Folin Ciocalteau method, which uses gallic acid as the reference standard.

The results obtained in this study are according to previous reports where the *L. ferrea* species was found to be rich in the phenolic compounds of the tannin and flavonoid type and have already been identified in extracts of pods, bark, stem, and flowers of *L. ferrea* (11, 16). In addition to triterpenes and steroids, the following metabolites were found in the phytochemical screening of fresh leaves: saponins, organic acids, reducing sugars, phenols, and tannins (13, 22).

The presence of anti-inflammatory flavonoids and anti-septic tannins in the crude extract of Jucá enhances its wound healing properties. Flavonoids have great chemical diversity with different biological activities; however, their major contribution to the healing process is related to their anti-inflammatory and antioxidant properties, which help modulate inflammation and prevent the formation of reactive oxygen species produced by inflammatory stress (8). Unfortunately, financial and equipment limitations prevented further and more detailed chemical analysis, but our preliminary chemical identification is related to the biological activities described.

### Characterization of Phytopharmaceuticals

The phytopharmaceuticals formulated are macroscopically stable, with an appearance, color, and odor characteristic of Jucá. The topical phytopharmaceutical based on murumuru butter (PyM) exhibits in the PLM birefringence with streak textures, which is a characteristic of the hexagonal phase. Crystalline liquid systems of the hexagonal type can resist erosion by body fluids and increase the retention time of the formulation at the site of application, enabling a sustained release of drugs, and enhancing therapeutic efficacy (23). In the centrifugation test, the polymer chains in the PyC remained intact, without phase separation.

### Wound Healing in Dogs

**Figure 3:**
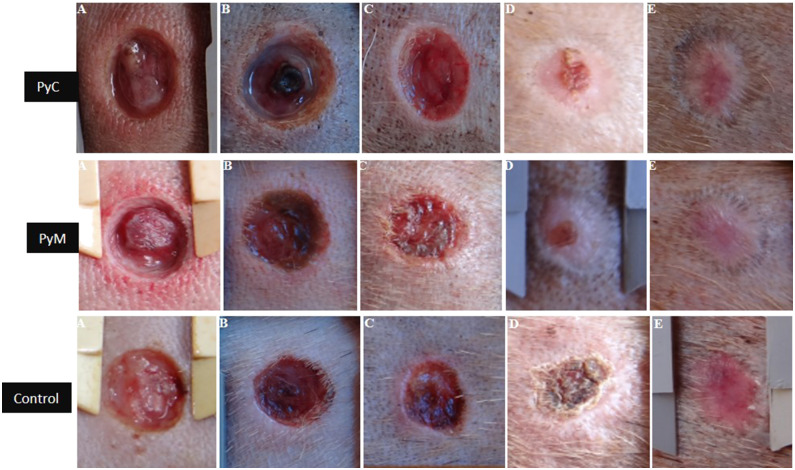
Macroscopic evolution of the dermal wounds. PyC: Carbopol based phytopharmaceutical; PyM: murumuru butter based phytopharmaceutical formulation; Control: commercial dermatological veterinary ointment. A: day 1; B: day 4; C: day 7; D: day 14; E: day 21.

In general, the dermal wounds healed satisfactorily in response to all treatments, with fully healed wounds visible by D14 (in the murumuru butter formulation and control groups). Overall, 91% of wounds were fully healed by D21. Figure 3 illustrates the wound healing evolution by treatment. The frequency of macroscopic alterations at each time-point are described in Table 2. Macroscopical evaluation of wounds treated with the PyC, PyM, and control formulations demonstrated the presence of serous exudate from D4 to D7. Serous exudate is related to the rupture of the lymphatic vessels, stimulating the release of the chemical mediators that trigger the inflammatory process (10). No differences were observed in the presence of exudate between the treatments at both D4 (*P* = 0.693) and D7 (*P* = 0.281). Seropurulent or purulent exudate were not present in any of the animals.

**Table 2:** Frequency of macroscopic alterations in standardized dermal wounds of dogs treated with a phytopharmaceutical based on murumuru butter (PyM), phytopharmaceutical based on Carbopol (PyC), and a commercial dermatological veterinary ointment a base allantoin (CO) throughout the experiment.

**Groups**	**Wound macroscopic evaluation**				
	**Hyperemia (%)**	**Exudate (%)**	**Edema (%)**	**Crusts formation (%)**	**Healing (%)**
**D4**					
CO	81.8	45.5	81.8	45.4	0
PyC	81.8	63.6	90.9	72.7	0
PyM	90.9	54.6	90.9	27.2	0
P^*^	0.790	0.693	0.752	0.100	0
**D7**					
CO	81.8	18.1	36.3	36.3	0
PyC	81.8	36.3	63.6	63.6	0
PyM	100.0	9.0	45.4	45.4	0
P^*^	0.320	0.281	0.428	0.428	0
**D14**					
CO	0	0	0	63.6	9.0
PyC	0	0	0	72.7	0.0
PyM	0	0	0	63.6	9.0
P^*^	0	0	0	0.873	-
**D21**					
CO	0	0	0	9.09	81.8
PyC	0	0	0	9.09	90.9
PyM	0	0	0	9.09	81.8
P^*^	0	0	0	1.00	0.790

* *Comparison of the frequencies of macroscopic changes between the treatments at each moment of evaluation by the χ2 test*.

Hyperemia and edema were observed in all experimental groups from D4 to D7 and no differences were observed in the frequency of these alterations between the treatments used (*P* > 0.05). Most of the wounds treated with Jucá developed crusts with reddish or brownish features, raised edges, and a rigid layer covering the lesion partially or entirely. Similar crust appearances were reported after wound treatment with Jucá (7, 10). Crust formation may be due to the high concentration of tannins in the plant that precipitate proteins in the damaged tissues, favoring the formation of a protective layer, which decreases the permeability and exudation of the wound and favors tissue repair (24). The presence of flies or larvae at the interior or the edges of the wounds was not observed at any of the time-points evaluated, and flies were commonly present at the study site. Previous reports (7) observed insect repellent properties with Jucá extracts at concentrations of 50%.

Table 3 presents the wound diameter in the different time-points and Table 4 shows the wound retraction data at time-points D14 and D21 in relation to D0. At the end of the study we observed an increase of wound retraction for the PyC (15.4% retraction increase) and PyM (16.2% increase) groups in relation to the control. Similar results of higher wound retraction were found using a powder formulation from Jucá bark (25).

**Table 3:** Diameter (mm) of external wound measurements of dogs treated with phytopharmaceutical based on murumuru butter (PyM), phytopharmaceutical based on Carbopol (PyC) or commercial dermatological veterinary ointment a base allantoin (CO).

**T**	**PyC**			**PyM**			**CO**		
	**Mean**	**SD**	**95% CI**	**Mean**	**SD**	**95% CI**	**Mean**	**SD**	**95% CI**
D0	101.6	9.4	(94.9–108.2)	101.5	9.9	(94.87–108.2)	102.7	12.7	(96.0–109.3)
D1	129.4	15.6	(117.7–141.2)	129.8	20.7	(118.07–141.5)	130.1	20.4	(118.3–141.8)
D4	128.3	23.6	(114.6–141.9)	123.9	18.6	(110.30–137.5)	125.3	23.6	(111.7–138.9)
D7	102.1	26.8	(86.1–118.1)	98.2	20.8	(82.30–114.2)	103.6	29.4	(87.6–119.5)
D14	52.5	10.4	(44.1–60.8)	56.7	15.7	(48.41–65.1)	62.2	13.9	(53.8–70.5)
D21	45.5	6.4	(40.4–50.7)	45.5	8.3	(40.41–50.7)	53.7	9.9	(48.6–58.8)

*T, times; SD, standard deviation; CI, 95% confidence interval*.

**Table 4:** Mean value of the wound retraction percentage (%) at day 14 (D14) and day 21 (D21) of post-surgical evolution of the groups treated with phytopharmaceutical based on murumuru butter (PyM), phytopharmaceutical based on Carbopol (PyC) or commercial dermatological veterinary ointment a base allantoin (CO).

**Times**	**PyC**			**PyM**			**CO**		
	**Mean**	**SD**	**95% CI**	**Mean**	**SD**	**95% CI**	**Mean**	**SD**	**95% CI**
D14	47.7	12.2	(39.1–56.3)	43.9	15.6	(35.3–52.5)	39.0	13.7	(30.4–47.6)
D21	54.7	8.0	(49.8–59.7)	55.1	6.7	(50.2–60.1)	47.4	9.1	(42.4–52.3)

*SD, standard deviation; CI, confidence interval*.

Table 5 presents the GLM results for wound diameter and wound retraction. The factors day, treatment, and animal were significative with an interaction observed only of animal^*^day and animal^*^treatment, but not for treatment^*^day. The model used fits our data with R^2^ of 97.6 and 96.7% for wound diameter and wound retraction, respectively. Interestingly, the animal factor affected the healing process, with a great variation. Those results support our choice for the methodological approach used to repeat all treatments in each animal. Dermal healing varies according to immune and nutritional status but genetic factors can also be associated (26). Furthermore, our study standardized the health and nutrition of all animals, and we used dogs from different genetic backgrounds. Studies using mongrel dogs should consider this high individual variation when addressing dermal wound healing.

**Table 5:** Generalized linear model for the response variables wound diameter and wound retraction and the predicted source of variation.

**Source**	**Wound diameter**			**Wound retraction**		
	**df**	**F**	***P***	**df**	**F**	***P***
Day	5	645.0	0.000	2	387.96	0.000
Treatment	2	4.2	0.018	2	4.95	0.012
Animal	10	39.9	0.000	10	22.07	0.000
Day*Treatment	10	1.3	0.226	4	1.18	0.332
Day*Animal	50	6.8	0.000	20	5.15	0.000
Treatment*Animal	20	5.5	0.000	20	2.98	0.002
Error	100			40		
Total	197			98		
*R*^2^	97.6%			96.7%		

*Df, degree of freedom; R^2^, coefficient of determination*.

Table 6 shows the statistical analysis using the Fisher LSD method, comparing the treatments used. The formulations with Jucá presented a slight superior healing activity than the control, demonstrated by lower wound diameter and higher wound retraction (*P* < 0.05), suggesting that this performance has a causal relationship with the astringent, antimicrobial, and anti-inflammatory effects of the tannins and flavonoids present in the plant (8, 27). Phenols and tannins were associated with drying, anti-inflammatory and healing properties (28). The flavonoids, also found in the *L. ferrea* extract, have a great chemical diversity with different biological activities, being the major contributor to the healing process related to their anti-inflammatory and antioxidant properties, which aid in the modulation of the inflammation process and helps in avoiding the formation of reactive oxygen species produced by inflammatory stress (8). Surgical wounds treated with 10% Jucá powder ointments in Wistar rats showed differences compared to the control group (25). In Wistar rats the ethanolic extract of Jucá at 12.5% produced a shorter retraction time of cutaneous wounds than 50%, indicating that the concentration of the extract may influence the treatment result (16). Conversely, a subsequent study with cattle evaluating the 5 and 10% concentrations of Jucá showed no differences in wound healing (20). Such discrepancies may be due to the agents used as the vehicle in the different studies.

**Table 6:** Statistical evaluation of wound diameter and wound retraction in dogs treated with phytopharmaceutical based on murumuru butter (PyM), phytopharmaceutical based on Carbopol (PyC) or commercial dermatological veterinary ointment a base allantoin (CO).

**Treatment**	**Wound diameter (mm)**			**Wound retraction (%)**		
	**Mean**	**SEM**		**Mean**	**SEM**	
PyC	93.3	4.6	B	33.7	5.5	A
PyM	92.6	4.4	B	34.0	4.7	A
CO	96.3	4.3	A	28.4	4.9	B

*Different letters in the same column mean significative difference through generalized linear model analyses of variance with Fisher Least Significance Difference method. SEM, standard error of mean; CI, confidence interval*.

Other plants from the Fabacea family have been reported to have wound healing activity (29) but Jucá is definitively the most important plant used in folk medicine for wound healing in the Lower Amazon. Here we enhance the traditional knowledge using a more appropriate vehicle for the plant molecules, producing an ointment that can be used both by animals and humans and without the pain-related problem of the folk formulation, since our ointments do not produce pain. Further studies are needed to understand the specific mechanism by which Jucá products tend to stimulate dermal healing in animals, requiring the identification of the specific compounds responsible for the biological activity. Herbal products tested in this controlled and standardized study performed better for wound healing than the expensive allantoin-based ointment (50 g is priced at US$14).

One limitation of the present study was the absence of one negative control group, treated with the ointment base, in order to evaluate the additional healing of the natural products. Our initial intention was to include this negative control group, but this was limited due to animal welfare concerns (i.e., leaving the animals with an untreated wound), and due to legal issues since the government facility where the animals were kept refused to accept animals untreated wounds. Additionally, considering our tropical environment, the untreated wound had great risk of larvae parasitism, which could harm the experiment.

### Microscopic Analysis

**Figure 4:**
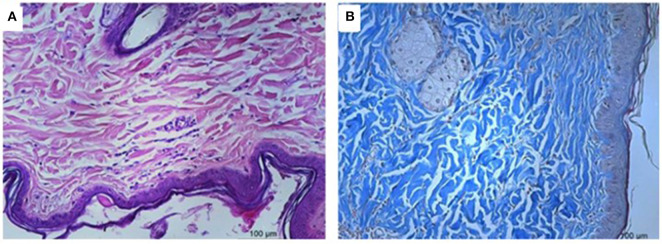
Microscopical evaluation of the dermal wounds at baseline (day 0). **(A)** Hematoxylin eosin staining; **(B)** Gomori trichrome staining.

**Figure 5:**
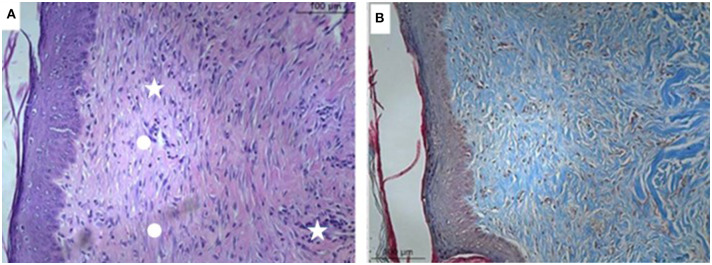
Microscopical evaluation of dermal wounds at day 21 from the PyC (Carbopol based phytopharmaceutical) group. **(A)** Hematoxylin eosin staining; **(B)** Gomori trichrome staining. Star indicates predominance of fibrocytes; circle indicate less dense collagen.

**Figure 6:**
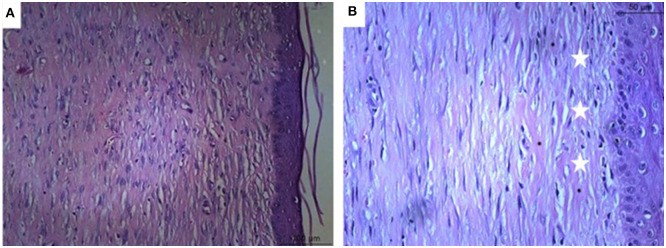
Microscopical evaluation of dermal wounds from the murumuru butter based phytopharmaceutical formulation (PyM) group at day 21. **(A)** Hematoxylin eosin staining; **(B)** Gomori trichrome staining. Star indicates collagen denser and polymerized.

**Figure 7:**
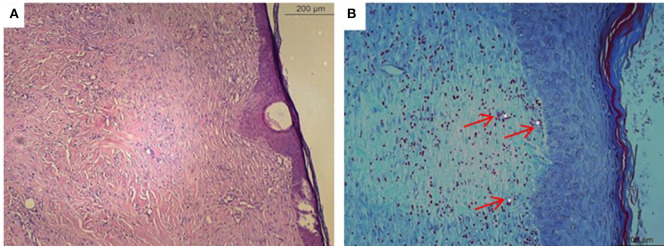
Microscopical evaluation of dermal wounds from the control group at day 21. **(A)** Hematoxylin eosin staining; **(B)** Gomori trichrome staining. Red arrows indicate angiogenesis.

Figure 4 presents the histological analysis of skin tissues at D21 compared to the baseline (D0). We observed differences in the epithelization process between the different treatments. In wounds treated with PyC (Figure 5), areas with epithelization and a prominent presence of fibroblasts were observed and the newly formed collagen showed good density and polymerization. In the wounds treated with PyM (Figure 6), we observed areas with epithelization, the presence of few fibroblasts, and predominance of fibrocytes; some vessels presented modeling and the collagen showed good density and polymerization, presenting an arrangement more organized than in the other treatments. In the wounds treated in the control (Figure 7), areas were observed with epithelialization devoid of adnexal elements and the presence of angiogenesis. The dermal reparative conjunctiva showed a similar proportion between fibroblasts and fibrocytes and a looser, less dense, and polymerized collagenous tissue.

In the histological evaluation, we observed treated wounds with both Carbopol and murumuru butter Jucá-based formulations to have a better epithelization process. Wounds treated with the herbal formulation had connective tissue with a large number of active fibroblasts, better organized collagen fibers, and few blood vessels, in contrast to the control group, where a moderate amount of fibroblasts, disorganized collagen fibers, and newformed vessels were observed, demonstrating the need for additional time to complete the epithelization process (26). Previous reports also demonstrated that the healing process was facilitated by phytotherapeutics with this plant (7, 25). Histopathological evaluation shows that the wounds treated with the Jucá-containing formulations had better healing resolution.

Besides the financial limitations of the present study, without further biochemical analysis such as hydroxyproline contents and additional histological staining, our results are robust enough to reach a conclusion. As far as we know this is the first study to evaluate Jucá for wound healing in dogs.

### Antimicrobial Activity

The ethanolic extract of jucá (*Libidibia ferrea*) exhibited antimicrobial activity against *S. aureus, E. coli, P. aeruginosa* and *C. krusei* by the MIC plate test, as observed in Table 7. The MIC for *Staphylococcus aureus, Escherichia coli, Pseudomonas aeruginosa*, and *Candida krusei* was 250, 125, 15.625, and 15.625 μg/ml, respectively. The crude jucá extract showed antimicrobial activity against *Candida krusei*, corresponding to a fungicidal action at a concentration of 500 μg/mL. Antibacterial activity against *Pseudomonas aeruginosa* was observed at 1,000 μg/mL. The reference antimicrobial drugs ciprofloxacin and nystatin had bactericidal and fungicidal activity, respectively, validating our results. Previous reports also demonstrated the antimicrobial activity of Jucá (10, 30). The antimicrobial activity found in this study is linked to the presence of tannins and flavonoids in the phytochemical analysis—both substances related to antimicrobial activity (31). The mechanism of antimicrobial action of tannins is related to inhibition of enzymes, modifying the metabolism at microbial membranes and decreasing the availability of essential ions for microbial metabolism (32).

**Table 7:** Antimicrobial activity of ethanolic extract of the jucá (*Libidibia ferrea*) at minimum inhibitory concentration test.

**Microorganisms**	**Ethanolic extract of the jucá (*****Libidibia ferrea*****) (μg/ml)**						
	**1,000**	**500**	**250**	**125**	**62.5**	**31.25**	**15.625**
*Staphylococcus aureus*	b	b	b	–	–	–	–
*Escherichia coli*	b	b	b	b	–	–	–
*Pseudomonas aeruginosa*	B	b	b	b	b	b	b
*Candida krusei*	F	F	f	F	f	f	f

*B, bactericidal activity; b, bacteriostatic; F, fungicidal; f, fungistatic*.

## Conclusion

The phytochemical study of the lyophilized extract of the pods of *L. ferrea* (Jucá) showed a high concentration of phenolic compounds and the presence of hydrolysable tannins and flavonoids.

Herbal formulations resulted in slightly better wound healing due to a smaller wound diameter and a higher wound retraction when compared with the commercial allantoin ointment. Jucá-based formulations contributed to dermal healing through wound fibroplasia. Jucá extract exhibited antimicrobial activity.

The results of this study suggest that herbal formulations containing 5% of Jucá ethanol extract have great potential for wound healing and can be used in the development of an herbal ointment for commercial veterinary use.

## Data Availability Statement

The datasets generated for this study are available on request to the corresponding author.

## Ethics Statement

The animal study was reviewed and approved by Committee on Ethics in the Use of Animals (CEUA/UFOPA). Protocol No. 0120180006.

## Author Contributions

ÁA, FA, SD, and CP conducted the field study with dogs, collected data, performed laboratorial analyses, and drafted the manuscript. KN and JA prepared and analyzed the ointments. KC and LB performed the chemical analysis. ACM performed histological evaluation and interpretation. SS performed antimicrobial activity tests and interpreted the results. AHHM designed the study and analyzed all the results. AHHM, SS, KN and LB revised the manuscript.

## Conflict of Interest

One formulation tested here used a crystalline liquid system (patent application BR 1020150308884) from one of the authors KN. The remaining authors declare that the research was conducted in the absence of any commercial or financial relationships that could be construed as a potential conflict of interest.
